# Stoichiometric capacitance reveals the theoretical capabilities of metabolic networks

**DOI:** 10.1093/bioinformatics/bts381

**Published:** 2012-09-03

**Authors:** Abdelhalim Larhlimi, Georg Basler, Sergio Grimbs, Joachim Selbig, Zoran Nikoloski

**Affiliations:** ^1^Institute of Biochemistry and Biology, University of Potsdam, Karl-Liebknecht-Str. 24-25, D-14476 Potsdam, Germany; ^2^Bioinformatics, Max-Planck Institute for Molecular Plant Physiology, Am Mühlenberg 1, D-14476 Potsdam, Germany; ^3^Systems Biology and Mathematical Modelling, Max-Planck Institute for Molecular Plant Physiology, Am Mühlenberg 1, D-14476 Potsdam, Germany

## Abstract

**Motivation:** Metabolic engineering aims at modulating the capabilities of metabolic networks by changing the activity of biochemical reactions. The existing constraint-based approaches for metabolic engineering have proven useful, but are limited only to reactions catalogued in various pathway databases.

**Results:** We consider the alternative of designing synthetic strategies which can be used not only to characterize the maximum theoretically possible product yield but also to engineer networks with optimal conversion capability by using a suitable biochemically feasible reaction called ‘stoichiometric capacitance’. In addition, we provide a theoretical solution for decomposing a given stoichiometric capacitance over a set of known enzymatic reactions. We determine the stoichiometric capacitance for genome-scale metabolic networks of 10 organisms from different kingdoms of life and examine its implications for the alterations in flux variability patterns. Our empirical findings suggest that the theoretical capacity of metabolic networks comes at a cost of dramatic system's changes.

**Contact:** larhlimi@mpimp-golm.mpg.de, or nikoloski@mpimp-golm.mpg.de

**Supplementary Information:** Supplementary tables are available at *Bioinformatics* online.

## 1 INTRODUCTION

Metabolic reactions play a fundamental role in sustaining cell growth through the import of nutrients from the environment and their conversion into molecules rendering a viable organism. Metabolic reactions do not operate in isolation; they are in fact related through shared metabolites and form large-scale metabolic networks (Klamt and Stelling, 2003; Schilling *et al.*, 2000; Schuster *et al.*, 2000). Altering the structure of metabolic networks provides the means for modulating their capabilities. To this end, metabolic engineering aims at re-programming cellular networks by controlling the activity of biochemical reactions in order to obtain a desirable output and has already found numerous application in biotechnology and medicine (Feist *et al.*, 2009; Lee *et al.*, 2011; Müller and Hausmann, 2011). From the early attempts of engineering bacterial strains, it had become apparent that the biosynthetic capabilities of organisms are limited not only by the input in a form of nutrients but also by the reaction fluxes obeying the basic physico-chemical constraints. Therefore, constraint-based approaches have gained considerable attention in the metabolic engineering community (Price *et al.*, 2003).

Based on the stoichiometry of the considered reactions, flux balance analysis (FBA) has provided the basic framework for investigating reaction fluxes in a metabolic network (Edwards and Palsson, 2000; Kauffman *et al.*, 2004; Lee *et al.*, 2006; Varma and Palsson, 1994). Extensions of this framework have subsequently allowed systematic investigations of network modifications directed at enhancing the production of selected targets. For instance, OptKnock (Burgard *et al.*, 2003), OptStrain (Pharkya *et al.*, 2004), OptReg (Pharkya and Maranas, 2006), OptForce (Ranganathan *et al.*, 2010) and CASOP (Haedicke and Klamt, 2010) as well as EMILiO (Yang *et al.*, 2011) have facilitated prediction and *in silico* verification of network modifications, including insertion and deletion of reactions from other species or under-and over-expression of gene products. All of these alternatives for network modifications rely on the assumption that gene expression is proportional to reaction flux. The findings of these optimization-based approaches suggest alternative pathways and/or flux redirections necessary to achieve the objective of interest. We note that the enumerated approaches, although very useful in designing strategies for metabolic engineering, are limited to the reactions already catalogued in various databases (Karp *et al.*, 2000; Kanehisa and Goto, 2000; Kumar *et al.*, 2012). The performance of the suggested strategies is in turn determined by the maximum achievable yield of a desired target which is calculated with the help of various optimization techniques.

Herein, we argue that quantifying the performance of metabolic engineering strategies in a given metabolic network should rely on the maximum ‘theoretically’ possible product yield in the network, given by the optimal inter-conversion of metabolites by using any biochemically feasible reaction. The notion of theoretical capacity refers to the estimated maximum production that can be delivered during a given period, provided a set of inputs. To emphasize the analogy, in the case of metabolic networks, the inputs are represented by the nutrients together with the set of metabolic intermediates and reactions, while the maximum production to be estimated is given by the suitably chosen objective function. Clearly, the inputs are given by the existing biochemical knowledge structured in a form of cellular networks together with the corresponding environmental constraints.

Estimation of the theoretical capacity for large-scale metabolic networks is all the more pressing issue due to the incompleteness of biological knowledge. The gaps in the current genome-scale metabolic networks arising as a result of the uncharacterized function of 20–60% of gene products (Hanson *et al.*, 2010) can have profound effect on the outcome of optimization-based approaches (Feist *et al.*, 2009; Marashi and Bockmayr, 2011). This provides the first justification for using the theoretical capacity as a golden standard. Therefore, the maximum theoretically possible yield lends itself as a standard analogous to the ‘theoretical capacity’ used in finance, transportation/logistics and batteries research (Todorov *et al.*, 1999; Transportation Research Board, 1997).

In addition, given the large fraction of unknown chemical reactions in biological systems and the immense space of macromolecules potentially catalyzing chemical reactions (Dobson, 2004), the most promising targets for metabolic engineering may not be found among the already characterized enzymes. Instead, synthetic alternatives obtained through systematic screening for chemically feasible, but so far uncharacterized, reactions may offer novel promising options for metabolic engineering (Basler *et al.*, 2012). This becomes particularly interesting in cases where the synthetic reactions can be readily decomposed into pathways of equivalent net conversion capability, involving only known enzymes.

The article is organized as follows: we first provide a brief overview of optimization-based approaches for metabolic network analysis. We then give a detailed and formal description of our novel concept, called stoichiometric capacitance. Our approach is designed to determine the theoretical capacity of a given metabolic network and relies on determining a feasible chemical reaction whose addition to the network results in maximum product yield. Therefore, it can be used in designing synthetic metabolic engineering strategies aimed at improving the yield of any product of interest. We illustrate the proposed concept on the Krebs cycle with the idea of verifying if glucose can be synthesized from fatty acids (de Figueiredo *et al.*, 2009). In addition, we apply the proposed method to improve biomass yield in genome-scale metabolic networks of 10 organisms from different kingdoms of life. Finally, we examine the implications of stoichiometric capacitance for the alterations in flux variability patterns.

## 2 METHODS

### 2.1 Optimization-based approaches

Metabolic networks are composed of metabolites inter-converted by a set of biochemical reactions. Based on the chosen system boundary, the metabolites in a given metabolic network can be categorized as ‘internal’ and ‘external’ (Heinrich and Schuster, 1996). The structure of a metabolic network is fully described by its stoichiometric matrix *S* ∈ ℝ^*m*×*n*^ which is equivalent to the incidence matrix of the underlying weighted directed hypergraph (Klamt *et al.*, 2009). The rows of the stoichiometric matrix correspond to the internal metabolites and each column represents a reaction. At steady state, the rate of formation of every internal metabolite equals the rate of its consumption. This is expressed by the flux balance equation
(1)
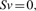

where *v*∈ ℝ^*n*^ denotes the vector of fluxes, also termed ‘flux distribution’, for the reactions included in the metabolic network. Aside from the flux balance conditions, flux distributions are also subject to other constraints related to thermodynamics, environmental conditions and flux capacities, which are often modeled as linear inequalities
(2)
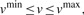

where *v*^min^ and *v*^max^ stand for the lower and upper bounds of the flux capacities of the considered reactions, respectively.

In addition, the law of mass conservation implies that all internal reactions must be stoichiometrically ‘mass balanced’, i.e. each stoichiometric column corresponding to an internal reaction must lie in the kernel of the mass matrix, *M*, which contains the molecular sum formulas of the internal metabolites. More formally,
(3)
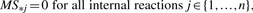

where *M*_*k,l*_ is the *k*-th atomic species (i.e. chemical element) in the sum formula of the *l*-th metabolite and S_*j_ stands for the *j*-th column in the stoichiometric matrix corresponding to the internal reaction *j*. Interestingly, this fundamental physical constraint is rarely considered in metabolic network analyses, although it provides a necessary precondition for the flux balance equation (1). Indeed, violation of equation (3) for any metabolite would allow for the removal or creation of atomic species out of nothing, thus rendering the flux balance equation meaningless.

The linear constraints given in equations (1) and (2) define a polyhedron
(4)
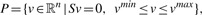

which contains all possible steady-state flux distributions. Here, we are interested in those flux distributions that maximize a predefined objective corresponding to a metabolic network function, such as growth or product yield. We assume that the network objective of interest is a linear function *τ* defined by a vector *c*∈ℝ^*n*^, such that *c*^*T*^ *v* ≥ 0 for all *v*∈*P*. As in FBA, the optimal value, τ*, for a metabolic network with stoichiometric matrix, *S*, is obtained by solving the following linear programming (LP) problem:
(5)
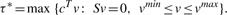


Clearly, the set of feasible fluxes for the problem in equation (5) is narrowed down by the constraints in equations (1) and (2). Namely, under these constraints, certain reversible reactions proceed only in one direction, while other reactions, termed ‘blocked’, are unable of carrying flux. A reaction carrying non-zero flux is termed ‘unblocked’. Furthermore, due to these constraints, a non-zero flux of certain reactions implies a non-zero flux through other ones (Burgard *et al.*, 2004). In particular, several reactions can be essential for performing the considered metabolic capability *τ*.

In analogy to essential reactions for the growth of a micro-organism (Edwards and Palsson, 2000), if τ* *>* 0, an unblocked reaction *i* is termed *essential* for *τ* if there exists *α*^*i*^ *>* 0 such that *c*^*T*^ *v* ≤ *α*^*i*^*v*_*i*_ for all *v*∈*P*. Let the essential reactions for *τ* be denoted by Ψ. If we have 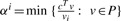 for all essential reactions *i*∈*Ψ*, then the optimal (i.e. maximum) capability τ* is given by
(6)
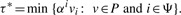

Therefore, increasing the maximum value of the objective τ* requires the relaxation of the upper bound imposed by the essential reactions on the objective *τ*. Such a relaxation either allows essential reactions to carry more flux or reduces the set of essential reactions, i.e. certain reactions are no longer essential for *τ*.

In the following, we introduce the concept of stoichiometric capacitance, which inherently depends on the aforementioned relaxation, and illustrate its applications in genome-scale metabolic networks as well as well-studied pathways.

### 2.2 Stoichiometric capacitance

The relaxation of some of the linear inequalities (2) may lead to an increase of the maximal objective τ* in the original network. This can be achieved by placing the living system in an environment which is in favor of the maximization of the objective *τ*; for instance, the living system can import more nutrients. The change in the objective value *τ* due to the relaxation of environmental conditions can be assessed using shadow prices in linear programming (Varma and Palsson, 1993). Alternatively, a significant improvement of *τ* can be obtained by determining a sparse vector *r*∈ℝ^*m*^ such that *τ* *(*r*)> τ* with
(7)
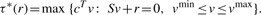

Let *I*(*r*)= {*i*∈{1,...,*m*} | *r*_*i*_ ≠ 0}. The vector *r* can be chosen to simulate a boundary reaction exporting (respectively importing) the accumulated (respectively depleted) internal metabolites in *I*(*r*). More importantly, if the vector *r* lies in the null space of the mass matrix *M*, defined in equation (3), then *r* represents the stoichiometry of a feasible chemical conversion of the internal metabolites *I*(*r*), up to multiplication by scalars. This may include any set of feasible chemical reactions with net conversion given by *r*. In the following, we refer to *r* as stoichiometric capacitance. We note that if the net conversion of the stoichiometric capacitance is thermodynamically feasible, its inclusion in the considered metabolic model, specified by equation (4), may increase the network objective, *τ*, given the same amount of nutrients.

In physics and engineering, the term capacitance is classically used to denote the change that must be added to a system to raise the electrical potential by one unit. Analogously, the concept of stoichiometric capacitance will denote the change in the metabolic structure given by the feasible chemical reaction whose inclusion in the network increases the objective of interest. The reference point for the increase is provided by the maximum value of the objective in the original metabolic network.

To investigate the extent to which we can improve the objective *τ* by adding a stoichiometric capacitance *r* to an investigated metabolic network, for each internal metabolite *i*∈{1,...,*m*}, we first introduce a binary variable *x*_*i*_ such that *i*∈*I*(*r*) implies *x*_*i*_ = 1. Let *μ* denote a given maximum number of non-zero components of *r* and *λ* be a predefined upper bound on the flux through the added reaction. The maximum capability τ*(*r*) and the corresponding stoichiometric capacitance *r* can be obtained by solving the following mixed integer linear programming (MILP) problem

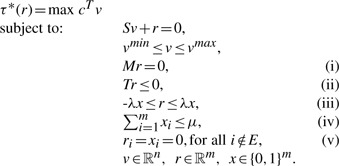

Condition (i) ensures that the stoichiometric capacitance, i.e. the added reaction, is chemically feasible. In other words, it ensures that *r* represents a mass-balanced net conversion that preserves the number of consumed and produced atoms. The inequality in (ii) guarantees that the stoichiometric capacitance is thermodynamically feasible, with *T* denoting the vector of the standard Gibbs free energy of formation of the internal metabolites. Constraints (iii) and (iv) ensure that *r* involves a limited number of metabolites from *I*(*r*), i.e. |*I*(*r*)| ≤ *μ*. Finally, the optional condition (v) allows modeling a stoichiometric capacitance that involves only a predefined subset *E* ⊆ {1,...,*m*} of metabolites. For instance, *E* may be the set of metabolites in a particular subcellular compartment or tissue. It is clear that further constraints can be included in the above MILP problem to determine a stoichiometric capacitance corresponding to a net conversion of only reactions that are more likely to occur in nature.

Although the stoichiometric capacitance represents a chemically feasible reaction, it may not correspond to any of the characterized enzymatic reactions from investigated organisms. Therefore, it is interesting to address the problem of determining if the stoichiometric capacitance, determined by solving the proposed MILP problem, can be expressed as a combination of a given set *ℛ* of known enzymatic reactions. The set *ℛ* can be defined by using the reactions catalogued in several existing databases of biochemical reactions (Kanehisa and Goto, 2000; Karp *et al.*, 2000; Kumar *et al.*, 2012).

Here, we define a linear programming problem whose solution resolves the raised issue. Suppose, the stoichiometry of the reactions in *ℛ*is given by the matrix

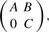

where *A*∈ℝ^*m*×;*p*^, *B*∈ℝ^*m*×;*q*^ and *C*∈ℝ^*m*′×;*q*^ such that *A* is a stoichiometric matrix defined in the space of *m* internal metabolites and 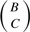 describes the stoichiometry of the reactions in *ℛ* which involve additional *m*^′^ metabolites. An identified stoichiometric capacitance *r* can be considered as a net conversion of the reactions in *ℛ* if there exist two vectors *α*∈ℝ^*p*^ and *β*∈ℝ^*q*^ such that

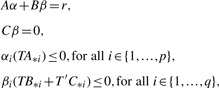

with *T*′ denoting the vector of the standard Gibbs free energy of formation of the additional *m*′ metabolites.

## 3 RESULTS AND DISCUSSION

We implemented the proposed method for determining the stoichiometric capacitance within the TOMLAB environment (Holmström, 1999).

**Fig. 1.**
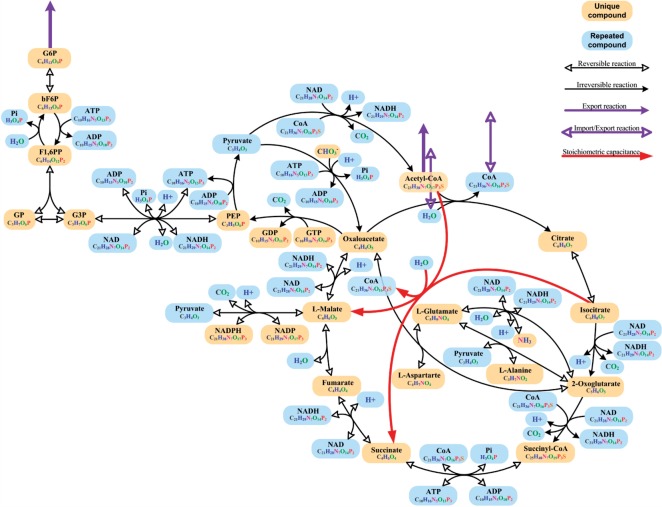
Metabolic model of glycolysis/gluconeogenesis and Krebs cycle in humans adopted from de Figueiredo *et al.* (2009). It has been shown that a net synthesis of glucose from fatty acids cannot be achieved by using the Krebs cycle. A stoichiometric capacitance with the synthesis of glucose as a metabolic function *τ* is colored in red. Adding this chemical reaction to the network allows for a net conversion of fatty acids into glucose

To illustrate our novel concept, we first consider the Krebs cycle whose mass-balanced reactions are depicted in Figure 1. Although a net synthesis of glucose from fatty acids may be feasible in the entire network of human metabolism (Kaleta *et al.*, 2011), a recent theoretical analysis has demonstrated that such a conversion cannot be achieved by using the Krebs cycle (de Figueiredo *et al.*, 2009). Here, we investigate whether this conversion becomes possible when a stoichiometric capacitance is added to the model of the Krebs cycle. To this end, we focus on finding a stoichiometric capacitance *r* with the objective of synthesizing glucose, where the set *I*(*r*) of involved metabolites consists only of the following six metabolites: acetyl coenzyme A (AcCoA), coenzyme A (CoA), isocitrate (Isocit), (S)-malate (Mal), succinate (Succ) and water (H_2_O). The rows of the matrices *M* and *T* corresponding to these metabolites are included in Supplementary Table S1.

Solving the proposed MILP problem, with *S* given by the stoichiometric matrix of the glycolysis/gluconeogenesis and Krebs cycle (de Figueiredo *et al.*, 2009), the matrices *M* and *T* for all the involved metabolites, and the synthesis of glucose as a metabolic function *τ*, results in the stoichiometric capacitance *r* whose equation is

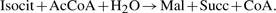

Interestingly, the stoichiometric capacitance *r* is the sum of the enzymatic reactions catalyzed by the isocitrate lyase (ICL) and the malate synthase (MAS) from the glyoxylate cycle:

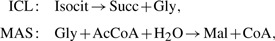

where ‘Gly’ stands for Glyoxylate. This result is in agreement with the findings in de Figueiredo *et al.* (2009) which state that glucose can be produced out of AcCoA using the Krebs cycle when the enzymes involved in the glyoxylate shunt are added. We note that the proposed MILP problem often does not have a unique solution, i.e. there may be more than one stoichiometric capacitance satisfying the imposed constraints. For instance, additional stoichiometric capacitances for the synthesis of glucose are given in Supplementary Table S2.

To benchmark the proposed method, in the following we explore the possibility of increasing biomass production in the following metabolic networks: *Saccharomyces cerevisiae*, *iND*750 (Duarte *et al.*, 2004), *Methanosarcina barkeri*, *iAF*692 (Feist *et al.*, 2006), *Mycobacterium tuberculosis*, *iNJ* 661 (Jamshidi and Palsson, 2007), *Escherichia coli*, *iJR*904, *iAF*1260, *iJO*1366 (Feist *et al.*, 2007; Orth *et al.*, 2011; Reed *et al.*, 2003), *Staphylococcus aureus*, *iSB*619 (Becker and Palsson, 2005), *Helicobacter pylori*, *iIT*341 (Thiele *et al.*, 2005), *Hordeum vulgare* seed (Grafahrend-Belau *et al.*, 2009) and *Bacillus subtilis* (Oh *et al.*, 2007). We constrain the stoichiometric capacitance to include up to four metabolites localized in the cytoplasm of the 10 considered networks. The results for the increase of biomass yield due to the inclusion of the stoichiometric capacitance are included in Table 1. It is evident that the stoichiometric capacitance allows for a very small increase of 0.75% in the case of *H. vulgare*'s relatively small and tightly constrained network. However, in the cases of *M. barkeri*, *iAF*692 and *E. coli*, *iAF*1260, the maximum theoretically possible yield is 160 847% and 3202%, respectively.

**Table 1.** Comparison of the increases in biomass yield by using the stoichiometric capacitance (SC)

Network	FBA	SC	Reaction equation
*M. barkeri*, *iAF*692	0.03	160847%	6 H_2_O + 2 nac (C_6_H_4_NO_2_) = 2 glycogen (C_6_H_10_O_5_) + N_2_
*S. cerevisiae*, *iND*750	0.10	72%	24 CO_2_ + 10 sbt_l (C_6_H_14_O_6_) = 14 glycogen (C_6_H_10_O_5_) + 19 O_2_
*M. tuberculosis*, *iNJ* 661	0.05	486%	11 H + 58 ppa (C_3_H_5_O_2_) = 22 glycogen (C_6_H_10_O_5_) + 3 ttdca (C_14_H_27_O_2_)
*E. coli*, *iJR*904	0.92	830%	12 CO_2_ + 20 H = 2 glycogen (C_6_H_10_O_5_) + 7 O_2_
*E. coli*, *iAF*1260	0.74	3202%	6 CO_2_ + 5 glc_d (C_6_H_12_O_6_) = 6 glycogen (C_6_H_10_O_5_) + 6 O_2_
*E. coli*, *iJO*1366	0.98	1714%	12 CO_2_ + 9 succ (C_4_H_4_O_4_) = 8 acon (C_6_H_3_O_6_) + 6 H_2_O_2_
*S. aureus*, *iSB*619	0.07	44%	5aizc (C_9_H_11_N_3_O_9_P) + arg_l (C_6_H_15_N_4_O_2_) = aicar (C_9_H_13_N_4_O_8_P) + citr_l (C_6_H_13_N_3_O_3_)
*H. pylori*, *iIT*341	0.69	370%	2 hom_l (C_4_H_9_NO_3_) = 2obut (C_4_H_5_O_3_) + acac (C_4_H_5_O_3_) + 2 NH_4_
*H. vulgare seeds*	2.76	0.75%	2 mal (C_4_H_6_O_5_) + 3 phpyr (C_3_H_5_O_7_P) = 5 CO_2_ + 3 e4p (C_4_H_9_O_7_P)
*B. subtilis*	0.40	58%	43 dha (C_3_H_6_O_3_) + 6 3dhsk (C_7_H_7_O_5_) + 9 pime (C_7_H_10_O_4_) = 39 glycogen (C_6_H_10_O_5_)

FBA stands for the optimal biomass yield using flux balance analysis, SC denotes the increases in the optimal biomass yield when a stoichiometric capacitance, determined by solving the proposed MILP problem, is added to the corresponding network. The last column shows the reaction equation of the corresponding stoichiometric capacitances.

**Fig. 2.**
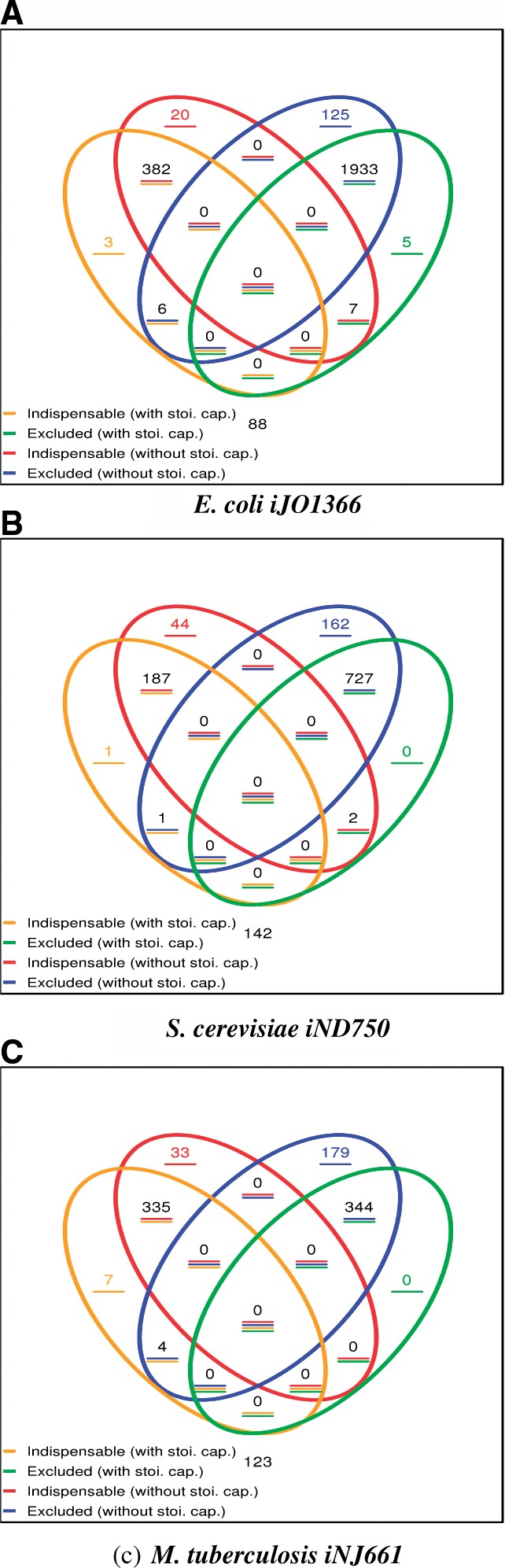
Venn diagram of reaction types in the metabolic networks of *E. coli*, *iJO*1366, *S. cerevisiae*, *iND*750 and *M. tuberculosis*, *iNJ* 661. The reaction types are determined by flux variability analysis using either the corresponding original FBA model (without stoi. cap.) or the altered FBA model (with stoi. cap.) which includes the corresponding stoichiometric capacitance given in Table 1. Excluded reactions are those which are not involved in any optimal FBA pathway, whereas reactions termed indispensable for growth carry non-zero flux in all optimal FBA pathways

To further investigate the effect of adding the stoichiometric capacitance to a given metabolic network, we used flux variability analysis (Mahadevan and Schilling, 2003). To this end, we computed the flux ranges with and without adding the corresponding stoichiometric capacitance to the networks of *E. coli*, *iJO*1366, *S. cerevisiae*, *iND*750 and *M. tuberculosis*, iNJ661. Reactions whose flux is zero in all optimal FBA pathways are called ‘excluded reactions’ as they are not involved in optimizing the biomass yield. Other reactions, termed ‘indispensable’ for growth, carry non-zero flux in all optimal FBA pathways. Figure 2 shows the changes in the types of reactions due the inclusion of the stoichiometric capacitance in the three considered network models.

By inspecting the panels in Figure 2, the following trends, obtained from the flux variability analysis, become apparent: (i) the number of indispensable reactions specific to the network with stoichiometric capacitance (in orange) is smaller compared to that of indispensable reactions specific to the original network (i.e. without the stoichiometric capacitance, in red), (ii) the number of excluded reactions specific to the network with stoichiometric capacitance (in green) is smaller compared to that of excluded reactions specific to the original network (in blue) and (iii) the number of reactions that switch from excluded in the original network to indispensable in the altered (in blue/yellow) is smaller than that of reactions that switch from indispensable in the original network to excluded in the network altered with the stoichiometric capacitance (in red/green); however, this holds valid only for *E. coli* and *S. cerevisiae*.

In addition, we observe in Figure 2 that only few reactions become indispensable (numbers in orange) or excluded (numbers in green) and only few reactions change between these types when adding the stoichiometric capacitance in the three networks. On the other hand, some of the indispensable (respectively excluded) reactions in the original network become no longer indispensable (respectively excluded). Consequently, the flux of these reactions may vary dramatically and so affect the optimal biomass yield, which corresponds to the relaxation of the constraints imposed on the feasible flux distributions when adding the stoichiometric capacitance. However, we point out that by far most reactions remain indispensable or excluded (red/orange and blue/green intersections), which demonstrates that the stoichiometric capacitance may still strongly rely on the fluxes of reactions from the original network, while allowing for a significant increase in biomass yield (Table 1). On one hand, this suggests that the identified stoichiometric capacitance allows for improving the performance of the original network without strongly redirecting the activity of the commonly used pathways, thus yielding a realistic scenario for metabolic engineering approaches. Nevertheless, since the changes happen across inspected types of reactions with and without stoichiometric capacitance, we conclude that the inclusion of the corresponding stoichiometric capacitance in each of the considered models may result in dramatic system's changes.

## 4 CONCLUSION

Our novel concept of stoichiometric capacitance allows not only for quantifying the theoretical capacity of metabolic networks with respect to a given objective but also suggests verifiable interventions (i.e. feasible chemical reactions) which provide this capacity, thus facilitating the design of metabolic engineering strategies. The used optimization-based formulation takes into account physico-chemical constraints to restrict the search space in a biochemically meaningful manner. To this end, we used a mixed integer linear program that can be efficiently resolved with existing optimization software. It becomes apparent that the proposed approach can easily be extended to consider non-linear objective functions discussed in Schuetz *et al.* (2007). Further constraints can be included to determine a stoichiometric capacitance corresponding to a net conversion of only reactions that are more likely to occur in nature. Our theoretical framework was used in addressing a concrete question that has long been debated in biochemistry, and concerns the possibility to produce sugars from fatty acids. The identified stoichiometric capacitance nicely confirms existing experimental results. This example also illustrates how the stoichiometric capacitance, taken as an overall reaction, can be decomposed into single enzymatic reactions. We also provided a linear program that addresses the issue of determining whether the proposed stoichiometric capacitance can be expressed as an overall conversion of a given set of enzymatic reactions. Furthermore, the analysis of genome-scale metabolic networks from various organisms showed that the stoichiometric capacitance could dramatically increase biomass production. Taken together, we believe that the presented approach will prove valuable not only in evaluating and classifying different metabolic networks according to their theoretical efficiency but also as a method of choice for designing synthetic metabolic engineering strategies.

*Funding:* A.L. is supported by the OPTIMAL project funded by the Federal Ministry of Education and Research, 0315958F. S.G. is supported by the ColoNET project funded by the Federal Ministry of Education and Research, 0315417F. Z.N. is supported by the Max-Planck Society.

*Conflict of Interest:* none declared.
